# Biomedical Applications of Lauric Acid: A Narrative Review

**DOI:** 10.7759/cureus.62770

**Published:** 2024-06-20

**Authors:** Ameena M, Meignana Arumugham I, Karthikeyan Ramalingam, Rajeshkumar Shanmugam

**Affiliations:** 1 Oral Pathology and Microbiology, Saveetha Dental College and Hospitals, Saveetha Institute of Medical and Technical Sciences, Saveetha University, Chennai, IND; 2 Public Health Dentistry, Saveetha Dental College and Hospitals, Saveetha Institute of Medical and Technical Sciences, Saveetha University, Chennai, IND; 3 Nanobiomedicine Lab, Centre for Global Health Research, Saveetha Medical College and Hospitals, Saveetha Institute of Medical and Technical Sciences, Chennai, IND

**Keywords:** anticancer agents, oral cavity cancer, oral diseases, antimicrobial, hypolipidemic, biomedical, antihypertensive, antitumour, coconut oil, lauric acid

## Abstract

Lauric acid, a major component of coconut oil, has been studied for its various health benefits over the years. Lauric acid is a medium-chained fatty acid with several potential biomedical applications based on its antimicrobial action, capacity for drug delivery, tissue engineering scaffolds, and cleansing capabilities. Various studies are carried out in vitro and in vivo using experimental animals, such as rats, shedding light on the efficacy of lauric acid. The studies related to lauric acid were brought under one umbrella and emphasized the need for further research to explore the efficacy of lauric acid in human health. This review aims to scientifically assess the reported data and present a narrative review on lauric acid in medicine.

## Introduction and background

Lauric acid (LA), also known as dodecanoic acid, a saturated fatty acid in coconut oil, is known for its potential health benefits and biomedical applications. Lauric acid accounts for approximately 56% of the triglycerides in coconut oil, which is the least explored for its biological potency [1]. Fatty acids like lauric acid are constituents of cell membranes. They are pivotal in cellular and biological activities, including transduction of signals, growth and differentiation, apoptosis, etc. [2]. Lauric acid is widely used in Ayurveda and contains polyphenolic antioxidants [1]. Lauric acid can also influence inflammation, metastasis, angiogenesis, tumor initiation, migration, and invasive nature [2].

It plays an essential role in different pathways of the central nervous system and cardiovascular system but its effects on other systems need to be explored. 

Chemical structure

**Figure 1:**
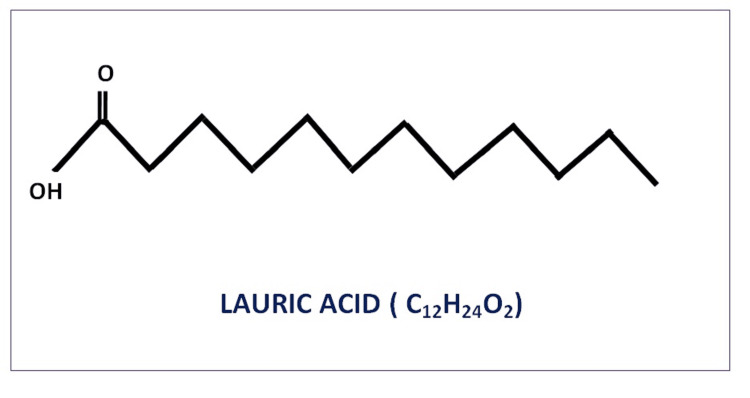
Lauric acid structure Image credit - Ameena M, Rajeshkumar S

LA is a 12-carbon atom (‘C12’) medium-chain fatty acid [3,4]. The chemical formula of LA is C_12_H_24_O_2_ and its molecular weight is 200.32 [5]. The main sources of LA are coconut and palm kernel oils and breastmilk [4,6]. Some edible insects like *Hermetia illucens* also contain lauric acid [4]. LA is the primary component of the diet in the tropical region, as coconut oil is widely consumed in these parts of the world, and LA accounts for 45-55% of coconut oil. The overall properties of LA are similar to those of capric acid (C10), as shown in various studies [7]. One hundred gm of coconut and hydrogenated coconut oil contain 41.8 and 44.2 gm of lauric acid, respectively [8]. The chemical structure of lauric acid is shown in Figure 1.

Commercially available lauric acid, pure C_12_H_24_O_2_- 99% (dodecanoic acid) is widely used in our labs.

Metabolism

**Figure 2:**
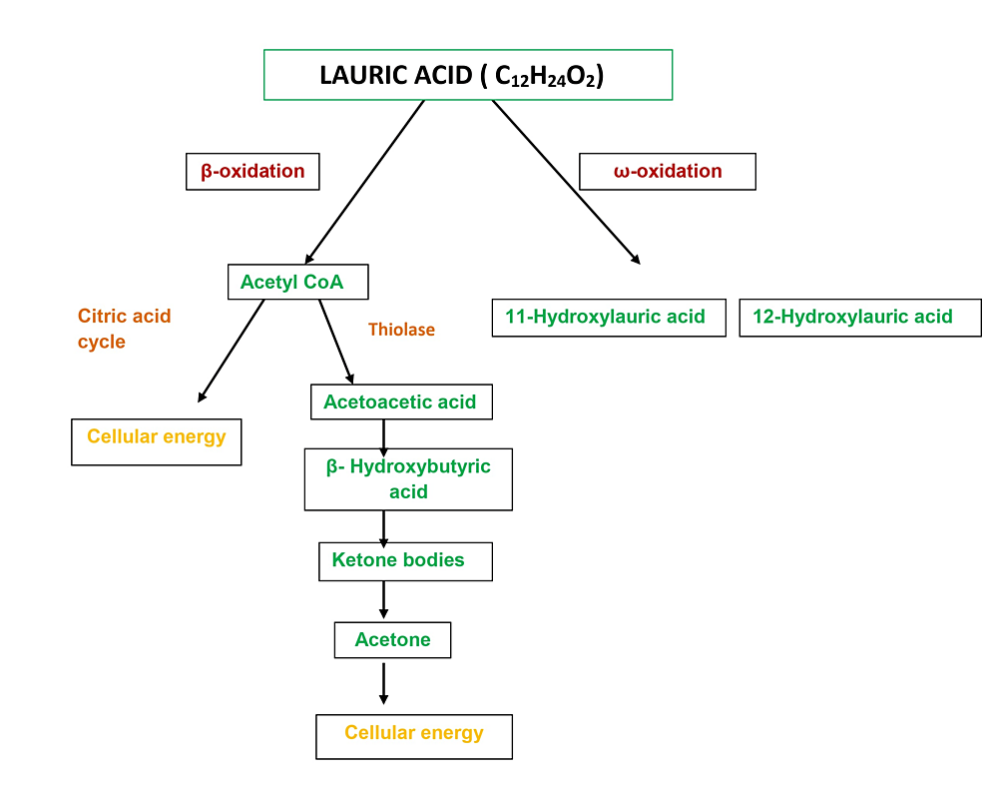
Metabolism of lauric acid

Medium-chain fatty acids, like LA, after being released from the triglyceride, are directly transported to the liver through the portal vein or resynthesized into new triglycerides that enter the lymphatic system [7]. It is promptly transported across the mitochondrial membrane by passive diffusion and oxidized by liver mitochondria by several mechanisms. Two acyl-coenzyme A dehydrogenase enzymes rapidly oxidize LA, and acetyl-coenzyme A production occurs. This increases ketone body production and develops a thermogenic response [7,9]. Instead of being stored in adipose tissue, lauric acid is metabolized to energy. Other byproducts, such as ketone bodies, are an immediate energy source for extrahepatic tissues like the brain, heart, and muscles [7]. The metabolism is shown in Figure 2.

Storage

The effects of lauric acid on serum cholesterol have given contradictory reports in various studies. The chances of fat accumulation and production of ectopic fat metabolites are low for LA as compared to other saturated fatty acids. Therefore, LA does not promote insulin resistance and inflammation [7].

## Review

Lauric acid is being studied for its antimicrobial, anti-inflammatory, and antitumor activity. It is also studied for its role in benign prostatic hyperplasia, hypertension, and diabetes as a drug to prevent secondary diabetic complications. It has also been explored for its cholesterol-lowering action, neuroinflammation, and Alzheimer's disease. It is also researched for ethanol-induced liver damage. Table 1 is the representation of biomedical applications using lauric acid.

**Table 1:** Various biomedical applications of lauric acid DPP-IV: dipeptidyl peptidase IV; HMG-CoA: hydroxymethylglutaryl-CoA; CDK: cyclin-dependent kinase; EGFR: epidermal growth factor receptor

Biomedical applications of lauric acid		
Implications in the nervous system	Prevent neuronal damage	Reduce the activation of microglial cells through the GPR40-dependent pathway
Cardiovascular implications	Reduce secondary coronary artery disease.	Reduce waist circumference and increase high-density lipoprotein cholesterol concentrations.
Anti-diabetic activity	Reduce secondary diabetic complications.	Induce inhibition of the aldose reductase enzyme and DPP-IV
Hypolipidemic activity	Inhibit cholesterol synthesis.	Reduce lipoprotein lipase and HMG-CoA reductase activity and lower the HMG/Mevalonate ratio.
Blood pressure control	Decrease the resistance of blood vessels	Alter Ca+2 channels and induces oxidative stress reduction in the kidney and heart.
Antitumor activity	Affect the viability of breast, endometrial, oral cancer cells, HepG2, and intestinal cells.	Increase expression of the CDK inhibitor p21Cip1/WAF1, through the PI3K/AKT pathway, downregulation of EGFR signaling, and cell apoptosis
Liver protection	Protect from ethanol-induced liver toxicity.	Neutralize superoxides and prevent lipid peroxidation and antioxidant depletion
Protection against benign prostatic hyperplasia	Reduce testosterone-induced hyperplasia	Reduce the prostate weight and downregulate the associated markers
Prevention of neuroinflammation	Maintain neural health	Maintain the cellular redox balance and mitochondrial health
Antimicrobial activity	Broad-spectrum antimicrobial activity	Cause membrane lysis by increasing cellular permeability

Antimicrobial activity

Lauric acid, one of the saturated fatty acids found to be the most inhibitory against Gram-positive bacteria, and its monoglyceride derivative of LA, glycerol monolaurate (GML), also shows significant antimicrobial activity [4]. Due to the broad-spectrum anti-microbial activity of LA, it can be used to control the balance of human gut microbiota [10]. It can also inhibit several fungal organisms and viruses [7]. Due to their amphipathic properties, medium-chain fatty acids like LA cause membrane lysis by increasing cellular permeability [11]. Bacterial cells inhibit the critical energy production processes by binding to electron carriers or interfering with oxidative phosphorylation, disrupting the electron transport chain. Another mechanism that explains the antimicrobial activity of MCFA is inhibiting membrane enzymes, such as glucosyltransferase, and other membrane-associated proteins [11]. LA can uniquely overcome microbial resistance because of its multiple antibacterial mechanisms and interference with cell signaling and transcription [7].

LA was found to be a persister cell inhibitor, consistently decreasing persister cell formation in the BW25113 *Escherichia* *(E.) coli* strain by 58-fold in a study by Jin et al. [5]. The high commercial value of LA can be explained based on its use as a precursor for antibacterial pharmaceutical products like mono-laurin [9]. Nowadays, several antimicrobial products are in the market using this property of LA. Insect-derived LA, derived from *Hermetia illucens*, is found to have antimicrobial resistance. The oil obtained from this insect might be antimicrobial, anti-inflammation, wound healing, and maintain gut microflora [4].

Nakatsuji et al. studied the role of LA in acne treatment as an antibiotic against *Propionibacterium acnes*, which is the causative organism for folliculitis or inflammatory acne [12]. They evaluated lauric acid's in-vitro and in-vivo antimicrobial properties against *Propionibacterium* *(P.) acnes*. They reported the minimal inhibitory concentration as 15 times lower than benzoyl peroxide when incubated with *P. acnes*, *Staphylococcus (S.) aureus*, and *S. epidermidis* with lauric acid, suggesting it has more potent antimicrobial properties than benzoyl peroxide. *P. acnes* was the most sensitive to lauric acid when the half-maximal effective concentration (EC50) was considered. They also found lauric acid was nontoxic to human sebocytes. The in-vivo study found decreased colonization of *P. acnes* in mouse ears after epicutaneous application and intradermal injection of lauric acid, thereby relieving *P. acnes*-induced ear inflammation. Based on their results, they emphasized the potency of lauric acid in the treatment of acne vulgaris [12-15].

Protection against benign prostatic hyperplasia

Lauric acid plays a significant role in protecting against benign prostatic hyperplasia through 5α reductase inhibitory activity in rats, as reported by a study by Veeresh et al. [16]. They reported that LA reduced the prostate weight and markers of testosterone-induced hyperplasia of the prostrate in Male Wistar rats. They also reported a remarkable reduction in disruption of the prostate's architecture by lauric acid in a histopathological examination [16].

Role in nervous system

In a study using primary cultured rat microglia and mouse microglial cell lines, Nishimura et al. reported that lauric acid stimulated nitric oxide (NO) production and inducible NO synthase protein expression without causing cell death [17]. They also reported a decrease in the production of lipopolysaccharide-induced reactive oxygen species (ROS) and proinflammatory cytokine production, c-Jun N-terminal kinase, and phosphorylation of p38-mitogen-activated protein kinase. GW1100, an antagonist of G protein-coupled receptor 40, also a plasma membrane lauric acid receptor, significantly reversed the lauric acid-induced suppression of nitric oxide generation. GW1100 treatment also reversed the lauric acid-induced decrease in lipopolysaccharide-induced phagocytosis. Further, lauric acid affected amyloid-β-induced augmentation of phagocytosis. Lauric acid, the primary component of coconut oil, affects microglial activation through the G protein-coupled receptor (GPR40)-dependent pathway, reducing activation of glial cells and further neuronal damage in Alzheimer's disease. Dietary intake of coconut oil reduces cognitive defects [17].

Neuroinflammation

Dietary supplementation with coconut oil has an essential role in reducing neuroinflammation. Ramya et al. analyzed coconut oil's antioxidant status and effects on human neuroblastoma cells and found that crude coconut oil and virgin coconut oil treatments showed good mitochondrial health [18]. ROS, inflammatory genes, interleukin (IL) 6, inducible nitric oxide synthase (iNOS), tumor necrosis factor (TNFα), and essential oxidative stress response genes (GCLC, Nqo1, and HO-1) in human neuroblastoma SH-SY5Y cells. They concluded that virgin and crude coconut oil maintained neural health mainly by keeping the cellular redox balance. They found that this result maintains cellular redox balance in neuroblastoma cells. Gas chromatography/mass spectrometry (GC/MS) results showed that lauric acid content significantly differed and depended on various extraction processes. They evaluated the potency of LA in SH-SY5Y cells and found a dose-dependent effect on neuroblastoma cells. LA was found to downregulate the inflammatory genes and oxidative stress response genes at a half-maximal inhibitory concentration (IC50) concentration of 11.8 μM. They emphasized the ability of LA to inhibit neuroinflammation and its potent cellular antioxidant activity, which further protects the cells. They also verified the efficiency of LA in normal cell lines such as fibroblasts (L929) to cross-validate the results for ruling out the chances of false positivity. Various concentrations of LA showed high compatibility in L929 cells. They concluded that the potent antioxidant activity of coconut oil provided cellular protection. Their results advised adding virgin or crude coconut oil to the diet for a healthy lifestyle [18].

Role in cardiovascular disease

More randomized controlled trials and prospective studies dealing with the association of coconut oil with cardiovascular disorders or stroke are needed [8]. A diet rich in extra virgin coconut oil, a rich source of medium-chain fatty acid lauric acid, reduces waist circumference and increases high-density lipoprotein cholesterol concentrations, thereby aiding in secondary prevention for patients with coronary artery disease, as reported in a study by Cardoso et al. [19].

Role in diabetes

Aldose reductase through the polyol pathway mediates secondary diabetic complications, and various studies report the use of coconut oil to increase insulin sensitivity and induce hypoglycemia. Sheela et al. studied the role of coconut oil and its components in secondary diabetic complications by studying polyol pathways using silico analysis [1]. They reported the inhibition of the aldose reductase enzyme and dipeptidyl peptidase IV (DPP-IV) by lauric acid. They validated the results in Wistar rats and confirmed the inhibition of enzymes involved in the process. There was a significant decrease in aldose reductase enzyme and sorbitol dehydrogenase activity in Wistar rats fed 1000 mg/kg of LA. In colorectal adenocarcinoma HCT-15 cells, there was a dose-dependent reduction in the aldose reductase enzyme. They concluded from the research that coconut oil and its components, like lauric acid, have a role in reducing secondary diabetic complications [1].

Lee et al. found that LA has the potential to induce nuclear factor (NFκB) activation and cyclo-oxygenase (COX-2) expression in the murine monocytic cell line RAW 264.7 [13]. McVeay et al. reported that intraduodenal administration of lauric acid with tryptophan reduced fasting plasma glucose in healthy men. They concluded that this effect is most likely by a mechanism involving both insulin and Glucagon‐like peptide (GLP-1) [14]. In another study, McVeay et al. found that lauric acid and leucine can stimulate gut hormones, especially cholecystokinin, and reduce energy intake in healthy men [15].

Anuar et al. studied the efficacy of LA in improving hormonal profiles, antioxidant activity, and sperm quality in infertile Sprague Dawley rats [20]. They also reported the alteration in the histomorphology of the testis and epididymis in LA-fed rats after injecting streptozotocin intravenously. They found a remarkable improvement in the fasting blood glucose level, glucose tolerance, and fertility after oral administration of LA. There was also a significant improvement in serum oxidative stress, epididymis, and testis. LA administration maintained the testicular and epididymal architecture, and there was also an improvement in sperm characteristics. They confirmed the role of LA in reducing hyperglycemia and maintaining insulin-glucose homeostasis [20].

In a study by Narayanankutty et al., they administered virgin coconut oil to male Wistar rats with a diet rich in fructose (60%) [21]. They reported a significant improvement in dyslipidemia and glucose metabolism. They found only a 17% increase in blood glucose levels in animals fed virgin coconut oil compared to copra oil-fed animals (46%). They could also see an improvement in hepatic redox status in the virgin coconut oil-fed group of animals compared to others. Histopathological examination revealed reduced steatosis and hepatic damage in virgin coconut oil. The results emphasized virgin coconut oil's efficiency in preventing calorie-rich diet-induced insulin resistance and secondary diabetic complications [21].

Hypolipidemic activity (cholesterol-lowering action)

The studies done on the Tokelauans population of the South Pacific and the Kitavan population of the Trobriand Islands group of Papua New Guinea show excellent cardiovascular health, even though they are the biggest consumers of coconut oil in the world. Despite studies that have reported the cholesterol-lowering activity of natural oils like coconut oil, there is always a controversy due to saturated fat's “artery-clogging” effect [22]. Lekshmi et al. used different target proteins to synthesize cholesterol using wet lab experiments and in-silico analyses to explore the hypolipidemic properties of coconut oil and its components [22]. They found lauric acid interacted more effectively with hydroxymethylglutaryl-CoA (HMG-CoA) reductase in the in-silico analysis. Molecular docking studies targeting different proteins like HMG-CoA reductase and cholesterol esterase showed the hypolipidemic potential of lauric acid. They also validated the results using male Sprague Dawley rats. Using a continuous spectrophotometric rate determination method, they found reduced lipoprotein lipase and HMG-CoA reductase activity in animals fed with LA. The HMG-CoA/mevalonate ratio was higher in lauric acid-treated groups than in control group animals and animals treated with atorvastatin, which shows the efficacy of lauric acid in inhibiting cholesterol synthesis [22].

Role in blood pressure control

Spontaneously hypertensive rats were used to study the effects on heart rate, blood pressure, and oxidative stress after acute administration of lauric acid by Alves et al. [23]. They reported a fall in blood pressure after intravenous doses of lauric acid in a dose-dependent fashion in Spontaneous hypertensive rats and Kyoto Wistar rats. They found a lauric acid-induced vasorelaxation in the superior mesenteric artery of Spontaneous hypertensive rats. They also reported reduced Nicotinamide Adenine Dinucleotide Phosphate Hydrogen (NADPH)-dependent superoxide accumulation in the kidney and heart. They concluded that LA could lower blood pressure in hypertensive and normotensive rats. They found that the hypotensive effect of LA could be due to the involvement of Ca+2 channels in the resistance blood vessels and its oxidative stress reduction potential in the kidneys and heart [23]. Alves et al. also reported a similar correlation between the oral supplementation with virgin coconut oil with exercise training and impaired baroreflex sensitivity, further lowering oxidative stress in spontaneously hypertensive rats [24].

Role in liver protection

Namachivayam et al. studied the effect of LA on ethanol-induced liver toxicity using albino rats [25]. They found a significant alleviation in inflammation, oxidative stress, lipid peroxidation, cell death, and increased expression of hepatocyte nuclear factor 4 (HNF4α) levels after administering LA orally. They also found a significant downregulation of pro-inflammatory cytokines and caspase-8/3 signaling. The normal histology was restored with upregulation of the B-cell lymphoma 2 (Bcl-2) and HNF4α. Thus, the study emphasized the role of LA in protecting the liver against ethanol-induced hepatotoxicity. They reported decreased antioxidant enzymes and malondialdehyde (MDA) levels due to LA. The oxidative damage brought on by ethanol overdose was significantly reduced by LA treatment. They confirmed the efficacy of LA in protecting against oxidative stress, neutralizing superoxides, and preventing lipid peroxidation and antioxidant depletion [25].

Antitumor activity

A study by Mustafa et al. used lauric acid in a nanogel combining chitosan and thiocolchicoside [26]. The nanogel was explored for its anticancer activity against oral cancer (KB-1) cells. They found that the nanogel with lauric acid had significant cytotoxicity to KB-1 cells [26]. LA affects the viability of SkBr3 breast cancer cells and Ishikawa endometrial cancer cells in a study by Lappano et al. [6]. They emphasized that the antiproliferative and cytotoxic action of LA is linked to the generation of ROS species, which involves transduction pathways in endometrial and breast cancer cells. They found an increased expression of the CDK inhibitor p21Cip1/WAF1 by treatment with LA, which was significant in its antitumor activity [6]. Verma et al. studied oncogenic and tumor suppressor miRNA in oral cancer cell lines KB cells and HepG2 cells after treatment with lauric acid using quantitative polymerase chain reaction (PCR) [2]. They used GeneMANIA software to assess the metabolic pathways of target oncogenes. They found a decreased expression of oncogenic miRNA and increased expression of some tumor suppressor miRNAs after treatment with LA [2]. An in-silico analysis was used by Buva et al., who tried to explore if the drug-likeness targets genes affected by LA, interactions of proteins, and hub genes, etc. [27]; they were able to find 23 common genes involved in cellular processes that play a crucial role in proliferation, programmed cell death, cell cycle regulation, and phosphoinositide 3‐kinase (PI3K)-protein kinase B (known as PKB or AKT) cascade. The 10 significant pathways include C-X-C motif chemokine ligand 8 (CXCL8), peroxisome proliferator-activated receptor-alpha (PPARA), matrix metalloproteinase (MMP9), androgen receptor (AR), and mitogen-activated protein kinases (MAPK1), and they highlight the role of lauric acid through the PI3K/AKT pathway in oral cancer treatment [27]. Sheela et al. explored the anticancer activity of LA using in silico tools, CDOCKER targeting proteins like epidermal growth factor receptor (EGFR), cyclin-dependent kinases (CDK), and thymidine synthase (TS), cell culture-based studies, and quantitative polymerase chain reaction [28]. They found anticancer activity of LA in human colon cancer (HCT-15 ), hepatocellular carcinoma cells (HepG2), and murine macrophages, Raw 264.7 cells in a dose-dependent manner. According to the study, EGFR expression was downregulated by 1.33 and 1.58 fold in HCT-15 cells when treated with 30 and 50 mg/mL of LA. The study thus concludes that the downregulation of EGFR signaling and cell apoptosis may account for its anticancer activity. They also reported morphological changes in LA-treated cells from lipid accumulation and cell membrane bulging to membrane blebbing and nuclear condensation. In higher concentrations, such as at 80µg/mL, the cells were shrunken and lost cellular integrity [28].

Fauser et al. studied the antitumor activity of lauric acid in intestinal epithelial cells (IEC-6) and p53-mutated colon cancer (Caco-2) cell apoptosis [29]. They reported a variation in glutathione and reactive oxygen species with LA. They also found a reduction of cells in the G0/G1 phase of the cell cycle and cell cycle arrest in the S and G2/M phases. Their study confirmed LA's antitumor activity in intestinal cell cells in the G0/G1 phase of the cell cycle and even at 0.5 and 1 mM concentrations induced IEC-6 cells [29]. Assiri et al. reported the anticancer and antioxidant potential of lauric acid-based hydrazones, namely, (E)- N′ -(naphthalene-1-yl methylene) dodecane hydrazide (NMDH), (E)-N′ -(2-nitro benzylidene) dodecane hydrazide (NBDH), and (E)-N′ -(4-fluoro benzylidene) dodecane hydrazide (FBDH) [30]. They found the greatest antioxidant activity with NBDH, and FBDH showed significant viability compared to others [30].

**Figure 3:**
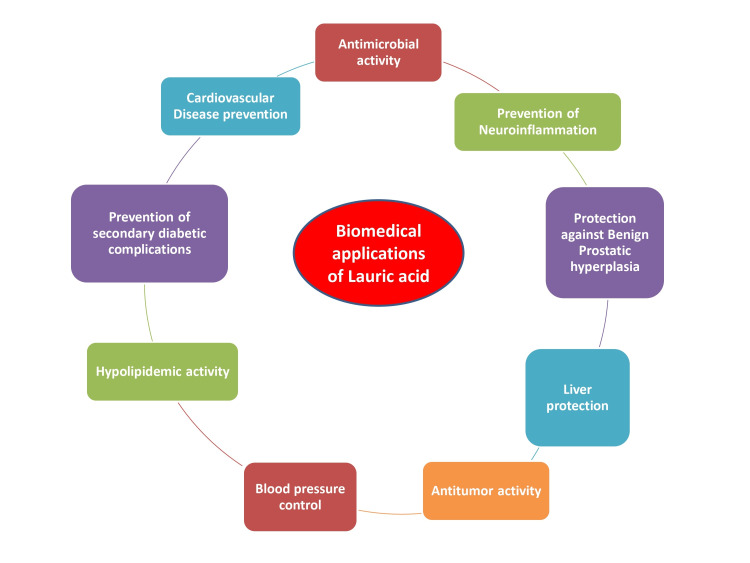
Biomedical applications of lauric acid

The biomedical applications of lauric acid are summarized in Figure 3.

Lauric acid formulations

Lauric acid was used along with thiocolchicoside and chitosan to develop a nanogel formulation by Ameena et al. [31]. It was explored for wound healing and cytocompatibility in human gingival fibroblasts. The prepared nanogel formulation with lauric acid showed excellent wound-healing activity and cell migration with nanogel-treated cells compared to the control group in an in-vitro scratch wound-healing assay. They revealed that the LA nanogel had a good cytocompatibility effect and increased cell viability of hGF cells at 4 0µl/ml concentration [31]. Another study by them used the same formulation and reported excellent antioxidant and anti-inflammatory properties of LA-containing nanogel. They found a percentage inhibition of 81% and 76% in bovine serum and egg albumin denaturation assay at 50 μg/mL concentration, respectively. They also reported the antimicrobial activity of the mentioned nanogel with a zone of inhibition of 20 mm and 22 mm at 100 µg/mL concentration against *Streptococcus mutans* and *Staphylococcus aureus, *respectively. The antioxidant activity of the prepared nanogel showed 89% and 81.6% inhibition in a 2,2-diphenyl-1-picrylhydrazyl (DPPH) and hydroxyl-free radical scavenging assay at 50 μg/ml [32].

Lauric acid in dentistry

We performed a PubMed search with keywords ("lauric acid"[All Fields]) AND ("dentistry"[All Fields]), and we were not able to find any articles related to dentistry. Research on the potential applications of lauric acid must be undertaken in dentistry, as it has good antimicrobial and antioxidant activity. Similarly, other possible applications in dentistry need to be explored.

Controversies and future scope

For decades, dietary recommendations demonized all fat, particularly saturated fat, for its supposed role in weight gain and disease. This perspective is undergoing a shift. New research suggests different types of saturated fat have distinct effects. Lauric acid, a medium-chain fatty acid abundant in coconut, is absorbed differently than those found in animal products and may even offer health benefits like improved cognitive function and a better cholesterol profile [33,34].

Oils rich in lauric acid can also have side effects on dyslipidemia, cardiovascular diseases, and general health. LA can interact with erythrocytes, affect its cellular membranes, and have marginal adherence properties that influence cardiovascular health. They can affect ion membrane transport and lead to calcium influx. LA can increase cholesterol levels and thereby impact the total cholesterol ratio. LA has been demonstrated to have the largest cholesterol-raising effect by increasing low-density lipoprotein-cholesterol and high-density lipoprotein-cholesterol. Free fatty acids are associated with an increased risk of sudden cardiac death, heart failure, and diabetes [18,33,34].

Traditionally viewed as a uniform category, saturated fats like lauric acid, found abundantly in coconut, are breaking the mold. Unlike their long-chain counterparts in animal products, medium-chain fatty acids like lauric acid are absorbed differently and show promise for improved cognitive function and a healthier cholesterol balance. This emerging research suggests the vital role of lauric acid, prompting a call to re-evaluate dietary recommendations and acknowledge the distinct metabolic effects of various saturated fats [33,34].

## Conclusions

The prevailing literature emphasizes the importance of restricting the intake of saturated fatty acids as a pivotal strategy in preventing cardiovascular disease. However, diverging from this narrative, numerous studies have emerged, shedding light on the potential benefits of coconut oil and its primary constituent of lauric acid. A significant gap persists in translating these in-vitro and in-vivo research observations to human subjects on a broader scale. Hence, there is an urgent need for further investigations to elucidate the utilization of lauric acid in larger human populations, especially in elucidating its indispensable functions within the cardiovascular and nervous systems. Moreover, additional research endeavors are warranted to delve deeper into the intricate cellular processes involving lauric acid. Understanding the precise mechanisms through which lauric acid operates holds promise for medical advancements and can potentially revolutionize therapeutic approaches. The multifaceted roles and potential applications of lauric acid could pave the way for a better understanding of health and disease and unravel innovative treatments and interventions within the medical realm.
